# Highly Active Amino-Fullerene Derivative-Modified TiO_2_ for Enhancing Formaldehyde Degradation Efficiency under Solar-Light Irradiation

**DOI:** 10.3390/nano12142366

**Published:** 2022-07-11

**Authors:** Jingbiao Fan, Tao Wang, Bo Wu, Chunru Wang

**Affiliations:** 1 Beijing National Laboratory for Molecular Sciences, Key Laboratory of Molecular Nanostructure and Nanotechnology, Institute of Chemistry, Chinese Academy of Sciences, Beijing 100190, China; fanjingbiao6277@163.com (J.F.); wangtaojk2007@163.com (T.W.); 2 University of Chinese Academy of Sciences, Beijing 100049, China

**Keywords:** amino-fullerene derivative, photocatalysis, chemical adsorption, HCHO degradation, TiO_2_ nanoparticle

## Abstract

Formaldehyde (HCHO) is a ubiquitous indoor pollutant that seriously endangers human health. The removal of formaldehyde effectively at room temperature has always been a challenging problem. Here, a kind of amino-fullerene derivative (C_60_-EDA)-modified titanium dioxide (C_60_-EDA/TiO_2_) was prepared by one-step hydrothermal method, which could degrade the formaldehyde under solar light irradiation at room temperature with high efficiency and stability. Importantly, the introduction of C_60_-EDA not only increases the adsorption of the free formaldehyde molecules but also improves the utilization of sunlight and suppresses photoelectron-hole recombination. The experimental results indicated that the C_60_-EDA/TiO_2_ nanoparticles exhibit much higher formaldehyde removal efficiency than carboxyl-fullerene-modified TiO_2_, pristine TiO_2_ nanoparticles, and almost all other reported formaldehyde catalysts especially in the aspect of the quality of formaldehyde that is treated by catalyst with unit mass (m*_HCHO_*/m*_catalyst_* = 40.85 mg/g), and the removal efficiency has kept more than 96% after 12 cycles. Finally, a potential formaldehyde degradation pathway was deduced based on the situ diffuse reflectance infrared Fourier transform spectrometry (DRIFTS) and reaction intermediates. This work provides some indications into the design and fabrication of the catalysts with excellent catalytic performances for HCHO removal at room temperature.

## 1. Introduction

Formaldehyde (HCHO) is a main pollutant in indoor environments, which can be absorbed by the human body through different channels and may be a serious harm to people’s health. The toxicity of formaldehyde is well-known and it is classified as a human carcinogen, which have made it an important risk to human health, even at low concentrations. It causes respiratory problems, leukemia, asthma, and other diseases [1,2,3,4]. Therefore, the effective removal of formaldehyde in the environment is a necessity [5,6,7,8,9]. Photocatalysis is an appropriate and promising technology for environmental applications, of which the absorption of photon energy from solar-light by photocatalysts can drive redox reactions of formaldehyde at room temperature [10,11,12,13]. Titanium dioxide (TiO_2_) is a kind of widely used photocatalytic material due to its excellent properties of low price, high activity, chemical stability, and nontoxicity [14,15,16,17]. However, as restricted by the wide bandgap (up to 3.2 eV) and the fast electrons and holes recombination rate [18,19].

The suitable particle size, optimized coating, and doping pure TiO_2_ with ions have always been the methods to increase the photocatalytic activity. Among the TiO_2_-supporting noble metals, Pt [20] and Au [21] have been regarded as the most active for formaldehyde degradation. However, the high price of noble metals greatly limits its large-scale application [22]. The unique physical and chemical properties of fullerenes have led many researchers to investigate the application of this molecule and its functionalized derivatives in various fields such as medicine, photovoltaics, gas adsorption/storage, and pharmaceuticals [12]. Many fullerene-semiconductor materials have been successfully developed for photocatalytic applications, such as TiO_2_/C_60_ [23], TiO_2_/C_70_ [24], ZnO/C_60_ [25], CdS/C_60_ [13,26], and C_3_N_4_/C_60_ [27,28]. These photocatalysts have been extensively studied for photocatalytic degradation of pollutants [29], disinfection [30], and water splitting for H_2_ evolution [13]. Typically, the C_60_-TiO_2_ nanocomposites have been reported to be able to efficiently catalyze degradation multiple organic substrates [12]. The incorporated C_60_ not only inhibits the charge recombination, but also broadens the light absorption range to a certain degree [23,26,30,31,32,33]. Nevertheless, the photocatalytic performance and the range of application of this kind of composites is still not satisfactory.

In pursuit of the high photocatalytic efficiency, the chemical adsorption plays an important role in formaldehyde removal. It has been reported that S- and N-containing functional groups (pyridines, amines, and sulfones/sulfonic acids) considerably enhanced the formaldehyde adsorption ability of carbonaceous adsorbents at ambient conditions [34,35]. Ma et al. [36] have synthesized an amino-functionalized graphene aerogel. As the carbonyl, carboxyl, and epoxy groups on the graphene layers were connected to the amino groups (-NH_2_), the molecular structure of HCHO was destroyed after being captured by the 3D structure of the aerogels. Significantly, the reaction of formaldehyde and -NH_2_ on the graphene sheets has dramatically promoted the catalytic activities.

Bearing the aforementioned in mind, herein, we have utilized amino-fullerene derivatives (C_60_-EDA) and TiO_2_ and constructed a novel nanocomposite for the first time. The introduction of the amino group has played a crucial role in improving the adsorption performance of materials through its specific binding with formaldehyde molecules [34,36,37,38,39], which was investigated by using in situ diffuse reflectance infrared Fourier transform spectrometry (DRIFTS). The obtained C_60_-EDA/TiO_2_ nanoparticles with a high utilization rate of sunlight and reinforced charge separation interaction, lead to enhanced photocatalytic degradation efficiency of formaldehyde.

## 2. Materials and Methods

### 2.1. Preparation of Photocatalysts

Tetrabutyl titanate (90 mL) was slowly poured into the aqueous solution. After continued stirring for 30 min, the mixed system was transferred to a 1 L reactor for hydrothermal reaction at 160 °C for 6 h. After the reaction was completed, the supernatant was removed and the crude products were centrifugally washed with deionized water and anhydrous ethanol for several times. Finally, the obtained products were air-dried at 60 °C for 12 h to obtain the pure TiO_2_ nanoparticles.

The synthesis of amino-fullerene derivatives (C_60_-EDA) and carboxyl-fullerene derivatives (C_60_-COOH) were followed the same procedures that were described in Ref. [40] and Ref. [41], respectively. Then, 212 mg C_60_-EDA or C_60_-COOH (mass ratio is 1 wt%) was dissolved into 450 mL deionized water to prepare an aqueous solution of fullerene derivatives, and 90 mL tetrabutyl titanate was slowly poured into the aqueous solution. After continued stirring for 30 min, the mixed system was transferred to a 1 L reactor for hydrothermal reaction at 160 °C for 6 h. After the reaction was completed, the supernatant was removed and the crude products were centrifugally washed with deionized water and anhydrous ethanol for several times. Finally, the obtained products were air-dried at 60 °C for 12 h to obtain the C_60_-EDA/TiO_2_ or C_60_-COOH/TiO_2_ composite.

### 2.2. Characterization of Photocatalysts

The morphology and structure of the samples were determined by scanning electron microscopy (JSM-6700F JEOL), energy dispersions spectroscopy (EDS), transmission electron microscopy (TEM), and high resolution TEM (HRTEM, JEM-2100F, JEOL). The phase structure of the composites was analyzed by X-ray diffractometer under 35 kV Cu Kα radiation source. Raman spectra were performed at room temperature with a 532 nm laser as the excitation source using Thermo Scientific spectrometer. BaSO_4_ was used as the reflectance standard material, and the UV-visible diffuse reflectance spectrum (DRS) of the material was measured by UV-visible diffuse reflectance spectrophotometer (UV-3100, Shimadzu Inc., Kyoto, Japan). The chemical states of the elements in the samples were analyzed by X-ray photoelectron spectroscopy (XPS, XSAM800, Kratos Analysis, Manchester, UK). Electron paramagnetic resonance (EPR) measurements were performed on a JEOL JE-FA200 spectrometer. Photochemical measurements were performed at an electrical workstation (CHI660E) using a conventional three-electrode system. Platinum wire and saturated calomel electrode (SCE) were used as counter electrode and reference electrode, respectively. The photocatalyst (5 mg) was dispersed in ethanol (0.5 mL) to obtain the grout, and then 20 μL of the grout was coated on ITO glass and dried overnight to obtain the working electrode. A 300 W xenon lamp with an AM 1.5 filter was used as the light source and 0.5 mol L^−1^ Na_2_SO_4_ solution as the electrolyte. The photocurrent response was recorded at a constant potential of 0.2 V. Electrochemical impedance spectroscopy (EIS) was performed at open circuit voltage, frequency of 0.05~10^5^ Hz, and amplitude of 5 mV. A Thermo Nicolet 6700 Fourier transform infrared (FTIR) spectrometer that was equipped with a mercury cadmium telluride (MCT) detector was used for the diffuse reflectance FTIR spectra measurements (DRIFTS). A praying mantis diffuse reflectance accessory and a reaction cell that was equipped with a heater (Harrick Scientific) were employed for the in situ sample treatment, photocatalytic reaction, and IR detection, as described in Refs. [42,43]. The TiO_2_ or C_60_-EDA/TiO_2_ powder samples, which were pressed into 7 mm × 1.5 mm cylindrical pellets (50 mg), were housed in a sample cup inside the reaction cell (Figure S1). Before the IR measurement, the samples that were filled in the sample cup were pretreated for 10 min under Ar flow (40 mL min^−1^, open switch valve I) to remove the adsorbed impurities, and the spectra of KBr were recorded for background removal. Subsequently, the O_2_ flow (10 mL min^−1^, open switch valve II) was bubbled through saturator containing formaldehyde, and then supplied to the reaction cell under dark conditions. The above process is called the HCHO adsorption process in the discussion of the paper, which was carried out for 70 min under dark, and the total gas flow rate was 50 mL/min (O_2_ flow: 10 mL min^−1^, Ar flow: 40 mL min^−1^). Finally, the O_2_ flow (10 mL min^−1^, open switch valve III and close switch valve II) was directly supplied to the reaction cell. Meanwhile, the catalysts were illuminated by a 300 W xenon lamp with an AM 1.5 filter for 60 min. The above process is called the HCHO oxidation process in the discussion of the paper, which was carried out for 60 min under light, and the total gas flow rate was 50 mL/min (O_2_ flow: 10 mL min^−1^, Ar flow: 40 mL min^−1^). All the spectra were recorded in the range of 650–4000 cm^−1^ by averaging 400 scans with a resolution of 4 cm^−1^ at scanning velocity of 20 kHz.

### 2.3. Photocatalytic Activity Test

The photocatalytic activity of the catalyst was tested in a homemade photoreactor (150 L, the length, width and height are 60 cm, 50 cm, and 50 cm, respectively), as shown in Figure S2. The reactor, formaldehyde detector, and gas chromatography were connected in series to form a closed-loop system. The gas in the reactor was mixed evenly and quickly through a fan. The peristaltic pump provided uniform flow power for formaldehyde in the reactor to ensure the stabilization of HCHO in the reactor and good contact between all the participants of the reaction system. Before the test, a Petri dish with a diameter of 15 cm was placed at the bottom of the reactor, containing 0.5 g sample. Then, 50 μL HCHO solution (38%) was injected into the reactor, and the initial concentration of HCHO was adjusted to about 100 ppm by controlling the humidity in the reactor to be about 55% and the temperature to be about 28–30 °C under dark conditions. It’s worth noting that the reactor needs to stand in the dark for 1 h after the injection of formaldehyde solution before proceeding to the next step to ensure that an adsorption-desorption equilibrium is established between the catalyst and formaldehyde. When the concentration of HCHO in the reactor stabilized, the xenon lamp light source was turned on to illuminate the sample surface. The concentration of HCHO in the reactor was detected by HCHO online detector (PN-2000 sensor) in real time. Meanwhile, the CO_2_ that was generated during HCHO oxidation was monitored by gas chromatography in real-time. The increase of CO_2_ concentration (ppm, ΔCO_2_, which is the difference between CO_2_ concentrations at t reaction time and initial time) and the decrease of HCHO concentration were recorded to evaluate the adsorption and catalytic performance. The removal rate of HCHO was determined as:

(1) $$\mathrm{HCHO}\;\mathrm{removal}\;\mathrm{rate}\;\left(\%\right)=\frac{C_{0}-C}{\;C_{0}}100\%$$

where $C_{0}$ (ppm) is the initial equilibrium concentration of HCHO before the test, and C (ppm) is the final concentration of HCHO at the end of the test.

## 3. Results and Discussion

### 3.1. Morphology and Structure Characterization

**Figure 1:**
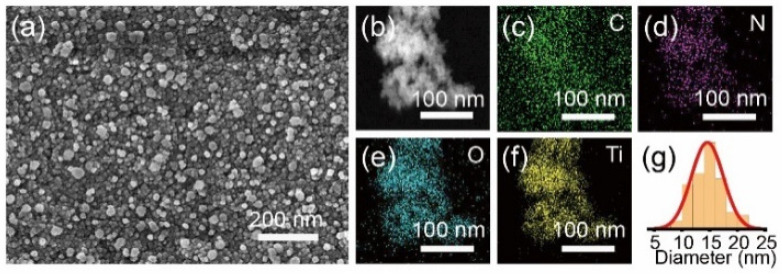
(**a**) SEM, (**b**) TEM, (**c**–**f**) EDS mapping, and (**g**) histogram of particle size distribution of C_60_-EDA/TiO_2_ composite.

**Figure 2:**
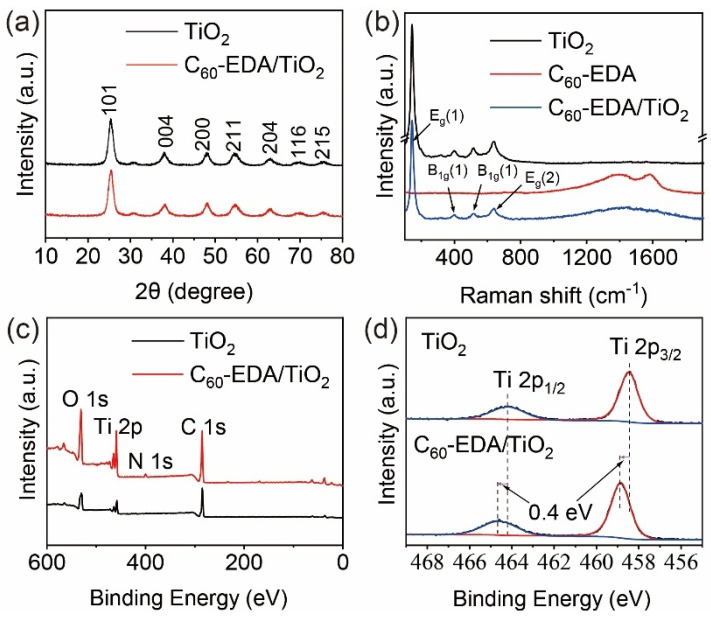
(**a**) XRD, (**b**) Raman, (**c**) full XPS, and (**d**) Ti 2p high−resolution spectra of pristine TiO_2_ and C_60_-EDA/TiO_2_.

The C_60_-EDA/TiO_2_ nanoparticles were synthesized using a surface-modification method. C_60_-EDA were immobilized on the surface of TiO_2_ nanoparticles in one step. The nanostructure of the sample was characterized by SEM and TEM. As shown in Figure 1a, the C_60_-EDA/TiO_2_ composite is composed of homogeneous nanoparticles with an average particle size of about 15 nm (Figure 1g). Compared with the pristine TiO_2_ nanoparticles (Figure S3 in the Supplementary Materials), the morphology of C_60_-EDA/TiO_2_ composite is also the particles, and the particle size are similar, indicating that the introduction of C_60_-EDA will not undermine the morphology of TiO_2_ nanoparticles. The TEM-EDS element distribution of C_60_-EDA/TiO_2_ nanoparticles are presented in Figure 1b–f, which clearly display the C, N, O, and Ti elements are uniformly dispersed. Moreover, the XRD pattern of the initial TiO_2_ nanoparticles was assigned to the typical structure of anatase phase [44] in Figure 2a. The peaks at 25.4° (101), 37.5° (004), 48.1° (200), 53.8° (105), 54.8° (211), and 62.8° (204) correspond to the pure anatase phase. After surface modification, the XRD pattern of C_60_-EDA/TiO_2_ composite display almost the same characteristic peaks as that of the pristine TiO_2_. The crystalline form of TiO_2_ nanoparticles remains virtually unchanged, coincident with the TEM results (Figure S4).

To further elucidate the interaction of C_60_-EDA and TiO_2_, Raman and XPS experiments measurements were carried out. As shown in Figure 2b, the peaks of C_60_-EDA/TiO_2_ at 144, 400, 515, and 636 cm^−1^ are the characteristic peaks of anatase TiO_2_ with Eg(1), B1g(1), A1g(1), B1g(2), and Eg(2) vibration modes [17,44,45]. The fullerene-related Raman peaks appear between 1200–1600 cm^−1^, which corresponds to the two typical peaks of C_60_-EDA (located at 1397 and 1579 cm^−1^), indicating the non-ignorable bond interaction between C_60_-EDA and TiO_2_ [17]. In the case of C_60_-EDA/TiO_2_ nanocomposites, N1s appeared in the XPS spectrum (Figure 2c), providing further evidence to the successfully introduce C_60_-EDA into the composite, which was consistent with TEM-EDS mapping results. At the same time, it can be observed that the positive displacement of Ti 2p binding energy of C_60_-EDA/TiO_2_ relative to that of TiO_2_ is 0.4 eV (Figure 2d), indicating that there is an electronic interaction between C_60_-EDA and TiO_2_ [45]. It is worth noting that compared with pristine C_60_-EDA, the binding energies of N-Hx (401.7 eV) and C-N (399.5 eV) in the N1s high-resolution spectra of C_60_-EDA/TiO_2_ (Figure S5) are positively shifted by 1.1 and 0.4 eV, respectively, which should be caused by the formation of N-O-Ti by the interaction of the amino groups on C_60_-EDA with the hydroxyl groups on the surface of TiO_2_ [16]. Electron-deficient Ti atoms tend to lower the electron density of N-O system via Ti-O bond, weakening the shielding effect of N valence electrons, thus increasing the binding energy of N1s [16,46,47].

### 3.2. Photocatalytic Properties

**Figure 3:**
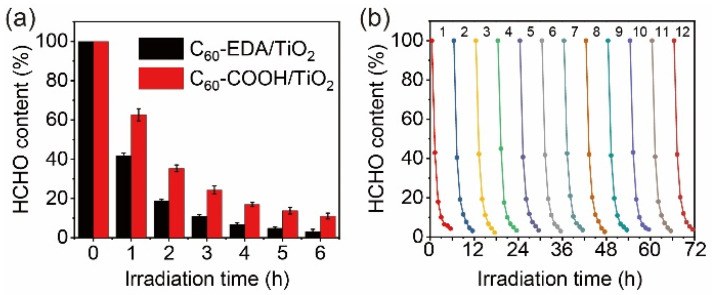
(**a**) Catalytic performance of C_60_-EDA/TiO_2_ and C_60_-COOH/TiO_2_ for the photodegradation of HCHO under simulated sunlight irradiation. (**b**) Cycling tests of C_60_-EDA/TiO_2_ for 12 cycles of photodegradation.

The photocatalytic formaldehyde degradation performance of C_60_-EDA/TiO_2_ composite was tested by a self-made photocatalytic apparatus (Figure S2). As shown in Figure 3a, the formaldehyde degradation rate of C_60_-EDA/TiO_2_ sample was about 97% after 6 h with simulated sunlight irradiation, which was significantly higher than that of C_60_-COOH/TiO_2_ (89%) and pristine TiO_2_ nanoparticles (32%; Figure S6). In comparison with the other reported formaldehyde catalysts [1,3,7,11,46,47,48,49,50,51,52,53,54,55,56,57,58] (Table S1), the catalytic activity of the C_60_-EDA/TiO_2_ composite is also significantly higher, especially in the aspect of the quality of formaldehyde that was treated by the catalyst with unit mass (m_HCHO_/_mcatalyst_ = 40.85 mg/g). It is worth noting that the degradation amount of formaldehyde and the generation amount of CO_2_ are close to 1:1, for example, the concentration of formaldehyde is reduced to 58 ppm after the degradation for 1 h, and the CO_2_ increase from 405 ppm to 462 ppm (Figure S7). Moreover, as shown in Figure 3a, the formaldehyde degradation rate of C_60_-EDA/TiO_2_ sample was 58% after 1 h, significantly higher than that of C_60_-COOH/TiO_2_ (38%). Therefore, the amino functional groups on the surface of the catalyst play an important role in the formaldehyde degradation progress. Among, the 1 wt% C_60_-EDA have achieved an optimal conversion efficiency (Figure S8).

What is more, the stability of the photocatalyst is another important factor for practical use. As shown in Figure 3b, there was no obvious decrease after 12 cycle tests in the catalytic activity for C_60_-EDA/TiO_2_. The HCHO degradation rate of C_60_-EDA/TiO_2_ sample were maintained in 96% after 6 h simulated sunlight irradiation in the 12th cycles, indicating the outstanding stability of the catalyst. The SEM and XRD analysis showed that the morphology and lattice structure of the samples did not change before and after the cycle tests (Figures S9 and S10).

### 3.3. Mechanisms of Formaldehyde Degradation

**Figure 4:**
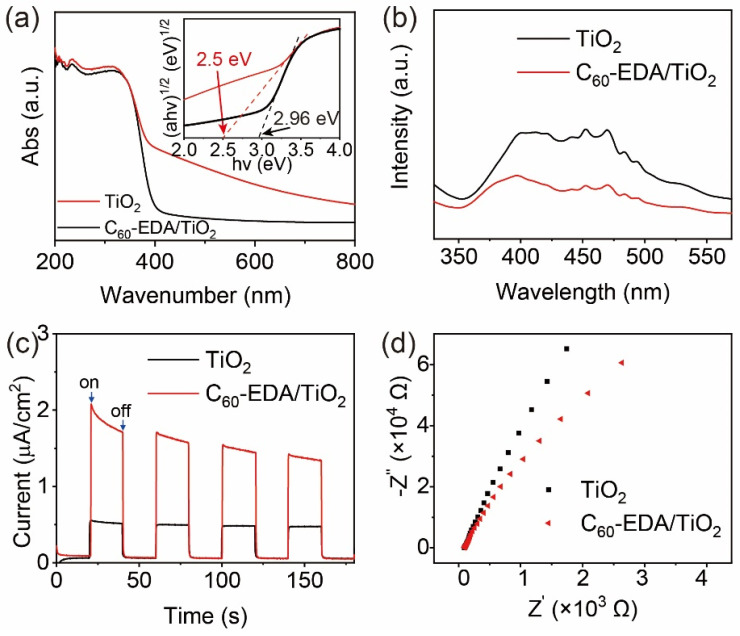
(**a**) UV diffuse reflectance spectra (inset is band gap energy diagram), (**b**) fluorescence spectra, (**c**) photocurrent response, and (**d**) AC impedance of TiO_2_ and C_60_-EDA/TiO_2_.

The light absorption property of the as-prepared photocatalyst was recorded in the diffuse reflectance spectra, as shown in Figure 4a. It can be seen that pristine TiO_2_ nanoparticles has almost no absorption above 400 nm, while C_60_-EDA/TiO_2_ expends the light absorption range obviously in the visible region. The band gaps of TiO_2_ and C_60_-EDA/TiO_2_ are 2.96 eV and 2.5 eV, calculated by the equation αhν = A(hν − Eg)^1/2^, where α, ν, Eg, and A are the absorption coefficient, frequency of light, band gap, and A constant, respectively [44,59]. Compared with pristine TiO_2_ nanoparticles, the C_60_-EDA/TiO_2_ composite has a higher efficiency for solar energy utilization, which makes a contribution to the improvement of the photocatalytic activity.

In order to explore the separation and recombination of photogenerated electrons and holes in the composite materials, the steady-state PL spectra of TiO_2_ and C_60_-EDA/TiO_2_ in the range of 350–550 nm were recorded under UV-light irradiation of 300 nm (Figure 4b). Compared with pristine TiO_2_, the PL emission intensity of the composite materials was significantly weaker. The results show that C_60_-EDA can be used as a good acceptor of photogenerated electrons in the sTiO_2_ conduction band and inhibits the recombination of photogenerated electrons and holes effectively [17,24].

To disclose the enhancing effect of C_60_-EDA on the photocatalytic activity, the photoelectrochemical properties of the C_60_-EDA/TiO_2_ nanocomposite were systematically examined. As can be seen from Figure 4c, the C_60_-EDA/TiO_2_ composite exhibits rapid photocurrent response under simulated solar irradiation, with a response intensity up to 2.0 μA/cm^2^, which is significantly higher than that of pristine TiO_2_ (0.5 μA/cm^2^), indicating that more photogenerated carriers can be effectively separated in the C_60_-EDA/TiO_2_ composite. It is shown that the introduction of C_60_-EDA increases the generation and transfer of charge carriers under sunlight, and effectively inhibits the recombination of photogenerated electrons and holes [17,60]. Further, the electron transport resistance of the C_60_-EDA/TiO_2_ photocatalyst was measured by EIS in Figure 4d. Generally, each arc in the plot represents a resistance during the charge transfer process, and a smaller radius correlates to a lower charge transfer resistance [17,60]. Accordingly, the radius of C_60_-EDA/TiO_2_ is significantly smaller than that of pristine TiO_2_, indicating that the charge transfer resistance of the composite material is lower and the charge separation is more effective. These findings perfectly coincide with the results that were obtained from the photo-response measurements.

Moreover, in order to identify the reactive radicals during the photocatalytic reaction process, in situ EPR measurements with DMPO as the spin trapping agent were performed. As displayed in Figure S11, the characteristic signals of hydroxyl radical (•OH) and superoxide radical (•O_2_^−^) were generated from the oxidation of H_2_O by the photogenerated holes, and the reduction of O_2_ by the photogenerated electrons [11,61,62,63]. The results of the EPR measurements proved that efficient separation of photogenerated electrons and holes of C_60_-EDA/TiO_2_ has produced more reactive oxygen radicals than TiO_2_ nanoparticles, so as to accelerate the reaction with formaldehyde.

**Figure 5:**
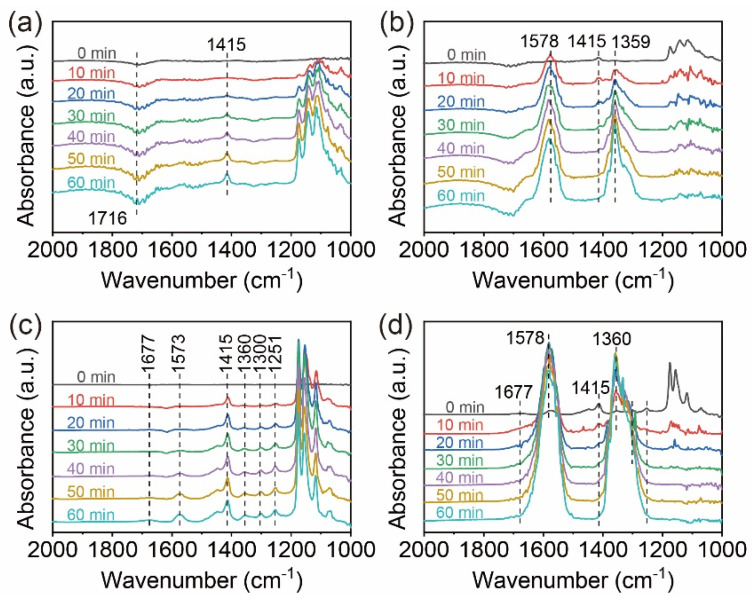
In situ DRIFT spectra of TiO_2_ (**a**) before and (**b**) after illumination; C_60_-EDA/TiO_2_ (**c**) before and (**d**) after illumination.

To clarify, the intermediate species of the catalysts in the reaction of formaldehyde at room temperature, in situ DRIFT spectra of the TiO_2_ and C_60_-EDA/TiO_2_ materials were performed (Figure 5 and Table 1). This mainly concerned the formaldehyde adsorption and oxidation process. According to previous studies, the bands that are located around 1000–1200 cm^−1^ ν(CO) and 1415 cm^−1^ δ(CH_2_) can be attributed to dioxymethyl (DOM) [6,7] and unstable hemiaminal [37,64]. The bands at 1578 cm^−1^ (ν_as_(COO)) and 1359 cm^−1^ (ν_s_(COO)) are attributed to formate and carbonate species, respectively [6]. In terms of pristine TiO_2_ (Figure S12), two negative bands at 3656 cm^−1^ ν(OH) and 1716 cm^−1^ δ(H-O-H) can be ascribed to the hydroxyl ν(-OH) and adsorbed water on the surface of TiO_2_ nanoparticle, respectively [6,43]. It is worth noting that the bands of C_60_-EDA/TiO_2_ at 1573 cm^−1^ ν(C=N) and 1360–1251 cm^−1^ ν(C-N) can be attributed to imines (C=N) and hemiaminal (-NH-CH_2_-OH) [34,36,37], indicating that the chemical absorption process of HCHO was realized based on the nucleophilic addition reaction between amine (-NH_2_) and aldehyde (-CHO) on the surface of the C_60_-EDA/TiO_2_ composites [35]. This process results in an exchange of protons to form an unstable hemiaminals which were subsequently dehydrated to Schiff base [37]. The band at 1677 cm^−1^ δ(H-O-H) can be ascribed to adsorbed water on the surface of the C_60_-EDA/TiO_2_ [6,7] which may be derived from unstable hemiaminal decomposition [35]. Therefore, the DOM, hemiaminal, Schiff base, formate, and carbonate are all the possible intermediate species during the process of formaldehyde adsorption and oxidation.

**Figure 6:**
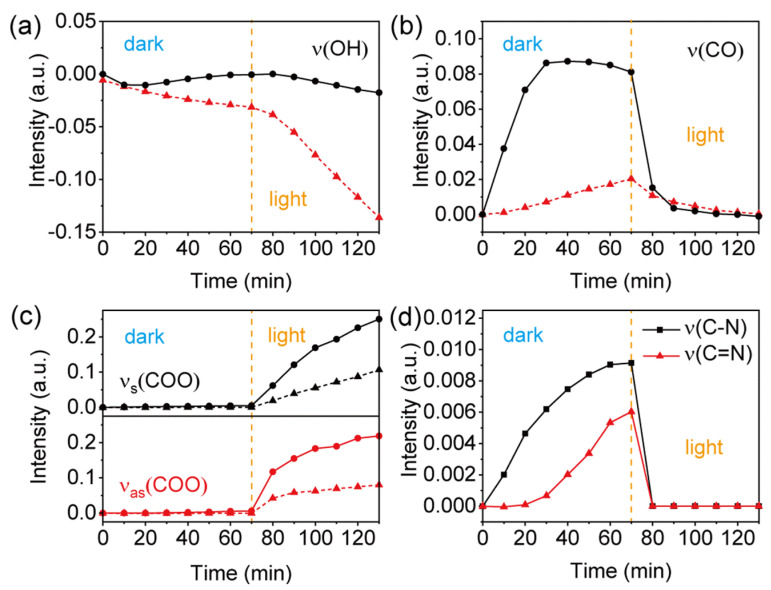
Peak intensities of surface species for formaldehyde adsorption and oxidation under dark (0~70 min) and light (70~130 min) as a function of the reaction time. (**a**) -OH species; (**b**) C-O species; (**c**) COO species; (**d**) C-N/C=N species. The solid line is for C_60_-EDA/TiO_2_ and the dashed line represents TiO_2_.

To analyze the contributions of C_60_-EDA and TiO_2_, the overall reaction of formaldehyde under dark or light on the surface of TiO_2_ and C_60_-EDA/TiO_2_ is performed. The featured band of ν(CO) was the product of the absorption of free HCHO molecules (Figure 6). In terms of the formaldehyde adsorption under dark (0~70 min), the peak intensity of ν(OH) weakened gradually, ν(CO) increased gradually, and ν_as_(COO)/ν_s_(COO) were nearly not observed for pristine TiO_2_ as the reaction took place (Figure 6a–c). It suggests that hydroxyl is gradually consumed and the HCHO that is adsorbed on the surface of the catalyst is converted into DOM [6,7]. It is worth noting that the peak intensity of the ν(CO) of C_60_-EDA/TiO_2_ is significantly higher than that of the pristine TiO_2_ (Figure 6b), but the peak intensity of ν(OH) does not decrease significantly in Figure 6a, indicating that the introduction of C_60_-EDA reduced the consumption of hydroxyl. The increased production of ν(CO) on the surface of the complex is due to the rapid chemisorption between the -NH_2_ and HCHO. In addition, the peak intensity of ν(CO) and ν(C-N) increased rapidly for C_60_-EDA/TiO_2_ during the reaction time of 0~30 min. Subsequently, the peak intensity of ν(CO) decreased slowly, ν(C-N) increased slowly, and ν(C=N) increased rapidly at the reaction time of 30~70 min; the band at 3511 cm^−1^ (adsorbed water) gradually increased in Figure S12c. It turns out that formaldehyde is adsorbed on the surface of the C_60_-EDA/TiO_2_ composite mainly through a reaction with the amino group. This process results in an exchange of protons to form an unstable hemiaminals which were subsequently dehydrated to form imines [37].

In terms of the reaction of formaldehyde oxidation of TiO_2_ and C_60_-EDA/TiO_2_ materials under light (70~130 min), the peak intensity of ν(CO) and ν(C-N/C=N) weakened rapidly (Figure 6b,d), and ν_as_(COO)/ν_s_(COO) gradually increased (Figure 6c), indicating that the light promotes oxidative decomposition of DOM and hemiaminal/Schiff base to formate/carbonate species. It is worth noting that the ν(C-N/C=N) basically disappeared completely after turning on the light source for 10 min (reaction time is 80 min) as shown in Figure 6d, while the peak intensity of ν(CO) of C_60_-EDA/TiO_2_ and TiO_2_ has a similar trend (Figure 6b), indicating that both DOM and hemiaminal/Schiff base existed on the surface of the C_60_-EDA/TiO_2_ composites, and hemiaminal/Schiff base is easier to oxidize to formate/carbonate species than DOM under light. Meanwhile, the peak intensity of ν_as_(COO)/ν_s_(COO) on the surface of C_60_-EDA/TiO_2_ composites is higher than that of pristine TiO_2_ in Figure 6c, which further proves this conjecture. The results show that amino on the surface of C_60_-EDA/TiO_2_ composites directly participates in the chemical adsorption and oxidation processes of HCHO.

**Scheme 1:**
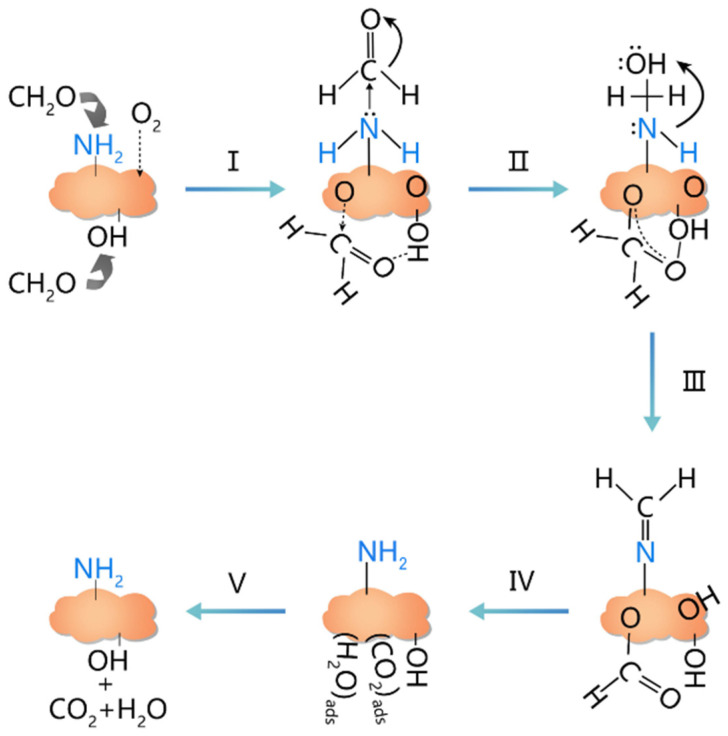
Schematic diagram of the possible pathway for photocatalytic oxidation of formaldehyde over C_60_-EDA/TiO_2_ under simulated sunlight.

Thus, a new insight into the reaction mechanism for the photocatalytic formaldehyde oxidation under simulated sunlight of C_60_-EDA/TiO_2_ has been proposed in Scheme 1. HCHO is adsorbed on the surface of C_60_-EDA/TiO_2_ composites by hydroxyl and amino group firstly (step I). Meanwhile, oxygen from air is adsorbed and splits into active oxygen species on the surface of C_60_-EDA/TiO_2_ composites. Then, the carbon (of carbonyl group) of HCHO is attacked by nucleophilic surface oxygen and active nitrogen atom on the surface of C_60_-EDA/TiO_2_ to form DOM and hemiaminal (step II). Subsequently, unstable hemiaminals are dehydrated to form Schiff base (step III). DOM is oxidized to formate under simulated sunlight irradiation (step III). Subsequently, the formate acid and Schiff base is further oxidized into adsorbed CO_2_ and H_2_O by active oxygen radicals on the composites surface (step IV). Finally, the adsorbed CO_2_ and water desorb from the composites surface, and hydroxyl and amino group active sites are regenerated again (step V). As we can see, the presence of C_60_-EDA promotes the adsorption and oxidation of formaldehyde and improves the photocatalytic activity of the C_60_-EDA/TiO_2_ composites (Figures S13–S17).

## 4. Conclusions

A novel C_60_-EDA/TiO_2_ nanocomposite was fabricated for the photocatalytic formaldehyde degradation efficiently. Comprehensive morphology studies along with surface and interface analyses have shed light on the interactions between C_60_-EDA and TiO_2_ nanoparticles, which play a vital role in the sunlight utilization and charge separation. The removal of formaldehyde should be divided into adsorption and catalytic oxidation processes. The exposed -NH_2_ active groups in the C_60_-EDA/TiO_2_ nanoparticles increased the adsorption ability of the formaldehyde molecule; some formaldehyde molecules combine with amino groups to form Schiff bases. Whereafter, the intermediate products Schiff base and DOM were oxidized to formate and carbonate by reactive oxygen on the surface, and then carbonate is decomposed into CO_2_ and H_2_O. The presence of C_60_-EDA plays an important role in the rapid adsorption of free formaldehyde molecules and increases the photocatalytic activity. Briefly, this work realizes high-rate photocatalysis formaldehyde degradation at room temperature, paving a new way for obtaining high-efficiency indoor formaldehyde removal at ambient conditions.

## Figures and Tables

**Table 1:** Infrared bands of surface species on TiO_2_ or C_60_-EDA/TiO_2_, Derived from Figure 5 and Figure S12.

Species	Assignment	Wavenumber (cm^−1^)	Refs.
DOM/Hemiaminal	ν(CO) δ(CH_2_)	1114, 1174 1415	[6,37]
Formate/Carbonate	ν_s_(COO) ν_as_(COO)	1359 1578	[6,7]
Hydroxy/Water	ν(OH) δ(OH)	3656, 3511, 3693 1716, 1677	[6,55,56]
Hemiaminal/Imine	ν(C-N) ν(C=N)	1251, 1300, 1360 1573	[34,36,37]

## Data Availability

The data presented in this study are available on a reasonable request from the corresponding author.

## Supplementary Materials

The following supporting information can be downloaded at: https://www.mdpi.com/article/10.3390/nano12142366/s1, Figure S1: Experimental apparatus for DRIFTS measurements; Figure S2: Schematic diagram of formaldehyde degradation device; Figure S3: SEM image of pristine TiO_2_ nanoparticles; Figure S4. HRTEM image of C_60_-EDA/TiO_2_ composites; Figure S5: N1s XPS spectra of C_60_-EDA and C_60_-EDA/TiO_2_ composite; Figure S6: Catalytic performance of pristine TiO_2_ nanoparticles for photodegradation of HCHO under simulated sunlight irradiation; Figure S7: Catalytic performance of C_60_-EDA/TiO_2_ for photodegradation of HCHO under simulated sunlight irradiation. Formaldehyde concentration and CO_2_ evolution amount with increasing photocatalytic time (Initial HCHO concentration at 100 ppm); Figure S8: Catalytic performance of the C_60_-EDA/TiO_2_ with different ratios of C_60_-EDA for photodegradation of HCHO under simulated sunlight irradiation; Figure S9: SEM images of the C_60_-EDA/TiO_2_ sample before and after 12 cycles of the photocatalytic reactions; Figure S10: XRD spectra of C_60_-EDA/TiO_2_ before and after 12 cycles of the photocatalytic reactions; Figure S11: EPR spectra in (a) methanol dispersion system and (b) deionized water dispersion system; Figure S12: In situ DRIFT spectra of TiO_2_ (a) before and (b) after illumination; C_60_-EDA/TiO_2_ (c) before and (d) after illumination; Table S1: Comparisons of the catalytic performances between C_60_-EDA/TiO_2_ and other reported formaldehyde catalysts; Figure S13. The molecular structures of the fullerene derivatives (a) C_60_-EDA and (b) C_60_-COOH; Figure S14. The XRD of TiO_2_ and C_60_-EDA/TiO_2_ with comparison with standard PDF card of anatase titanium dioxide; Figure S15. Catalytic performance without photocatalyst and with C_60_-EDA; Figure S16. Band gap energy diagram of (a) C_60_-EDA and (b) C_60_-COOH; Figure S17. XRD spectra of the C_60_-EDA/TiO_2_ with different ratios of C_60_-EDA.
