# Supplementary material for: Highly Active Amino-Fullerene Derivative-Modified TiO2 for Enhancing Formaldehyde Degradation Efficiency under Solar-Light Irradiation
Source: Nanomaterials (Basel). 2022 Jul 11;12(14):2366. doi: 10.3390/nano12142366 (PMC9321472; doi:10.3390/nano12142366)
Supplement: Supplementary file 1 [file nanomaterials-12-02366-s001.zip › nanomaterials-1814182-supplementary.pdf]

## Supplementary Materials

# Highly Active Amino-Fullerene Derivative-Modified $TiO_2$ for Enhancing Formaldehyde Degradation Efficiency under Solar-Light Irradiation

**Jingbiao Fan [1], Tao Wang [1,2], Bo Wu [1,2,*] and Chunru Wang [1,2,*]**

1. Beijing National Laboratory for Molecular Sciences, Key Laboratory of Molecular Nanostructure and Nanotechnology, Institute of Chemistry, Chinese Academy of Sciences, Beijing 100190, China; fanjingbiao6277@163.com (J.F.); wangtaojk2007@163.com (T.W.)
2. University of Chinese Academy of Sciences, Beijing 100049, China

* Correspondence: zkywubo@iccas.ac.cn (B.W.); crwang@iccas.ac.cn (C.W.)

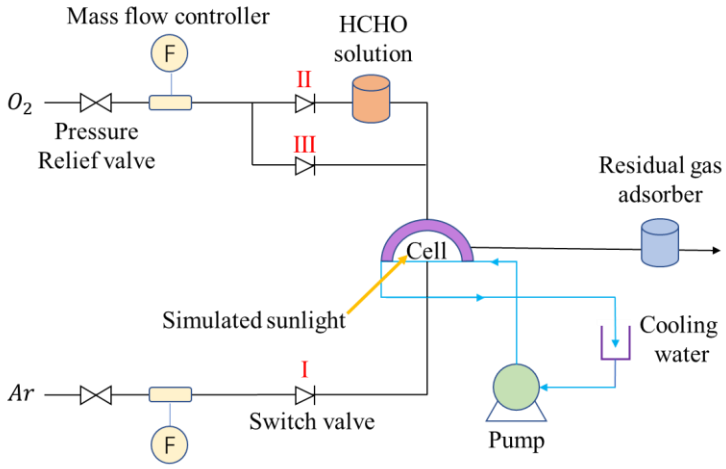


**Figure S1**. Experimental apparatus for DRIFTS measurements.

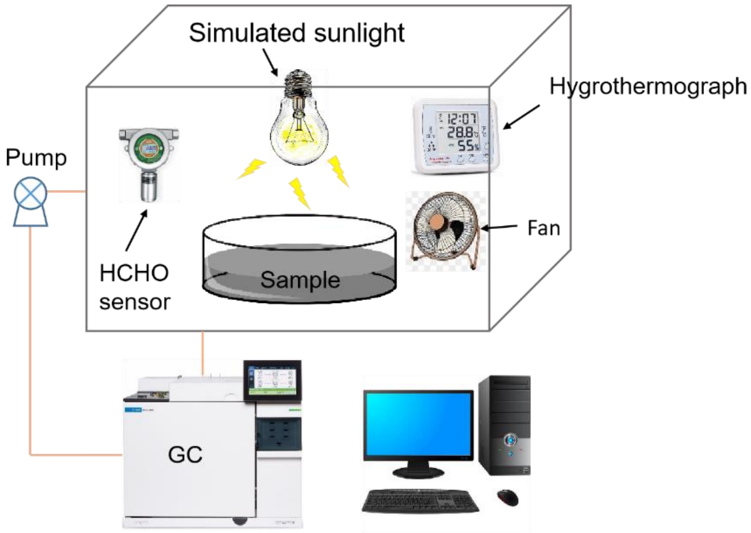


**Figure S2**. Schematic diagram of formaldehyde degradation device.

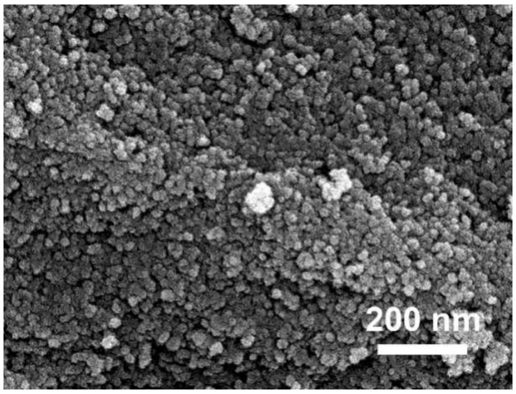


**Figure S3**. SEM image of pristine $TiO_2$ nanoparticles.

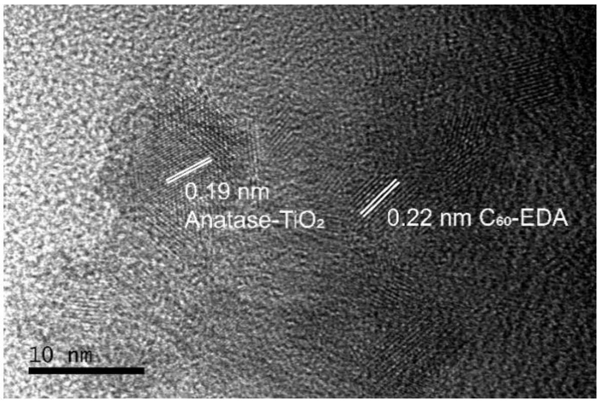


**Figure S4.** HRTEM image of $C_{60}$-EDA/$TiO_2$ composites.

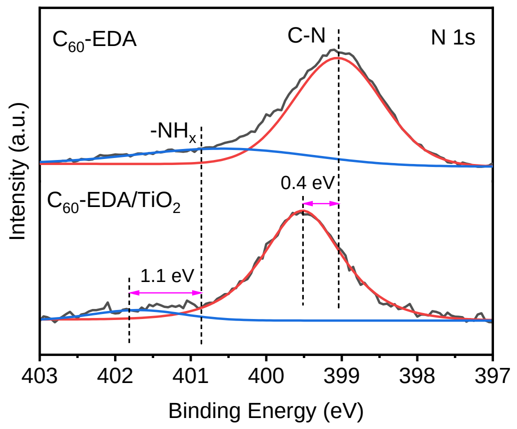


**Figure S5**. N1s XPS spectra of $C_{60}$-EDA and $C_{60}$-EDA/$TiO_2$ composite.

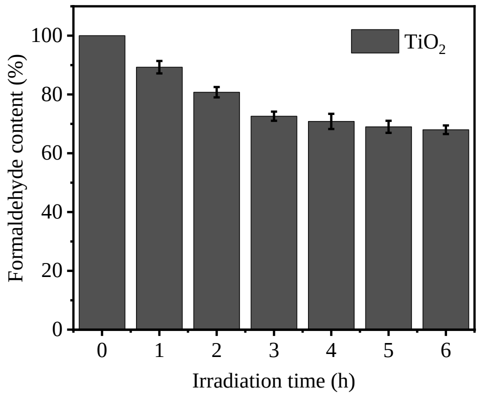


**Figure S6**. Catalytic performance of pristine $TiO_2$ nanoparticles for photodegradation of HCHO under simulated sunlight irradiation.

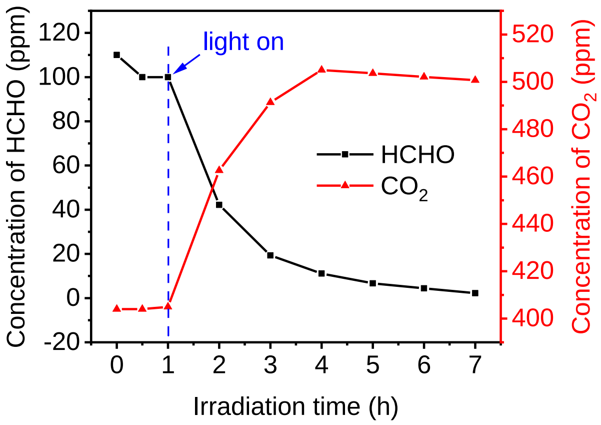


**Figure S7**. Catalytic performance of $C_{60}$-EDA/$TiO_2$ for photodegradation of HCHO under simulated sunlight irradiation. Formaldehyde concentration and $CO_2$ evolution amount with increasing photocatalytic time (Initial HCHO concentration at 100 ppm).

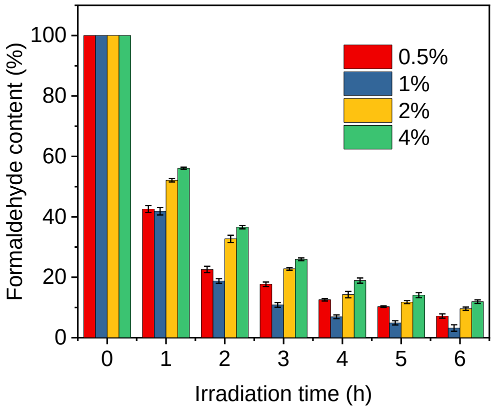


**Figure S8**. Catalytic performance of the $C_{60}$-EDA/$TiO_2$ with different ratios of $C_{60}$-EDA for photodegradation of HCHO under simulated sunlight irradiation.

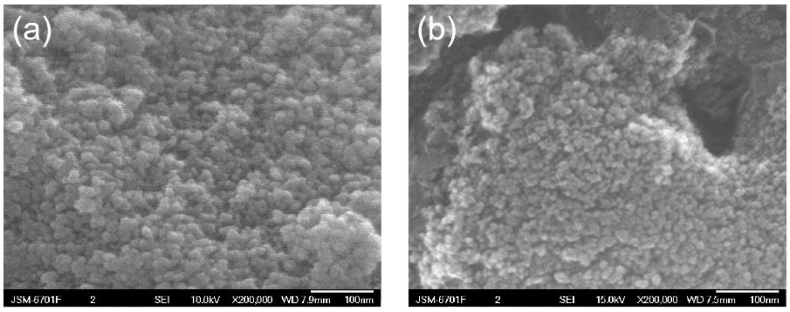


**Figure S9**. SEM images of the $C_{60}$-EDA/$TiO_2$ sample before (a) and after (b) 12 cycles of the photocatalytic reactions.

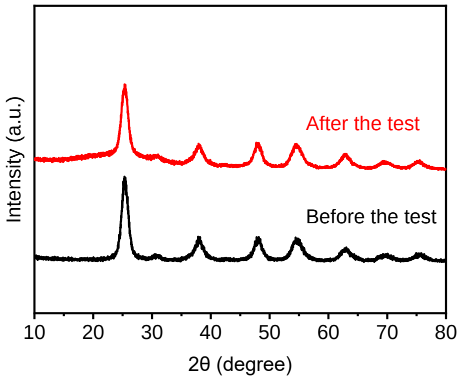


**Figure S10**. XRD spectra of $C_{60}$-EDA/$TiO_2$ before and after 12 cycles of the photocatalytic reactions.

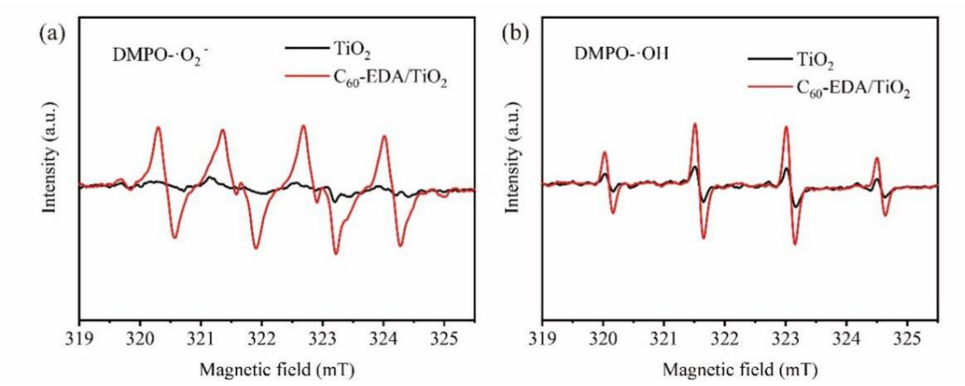


**Figure S11**. EPR spectra in (a) methanol dispersion system and (b) deionized water dispersion system.

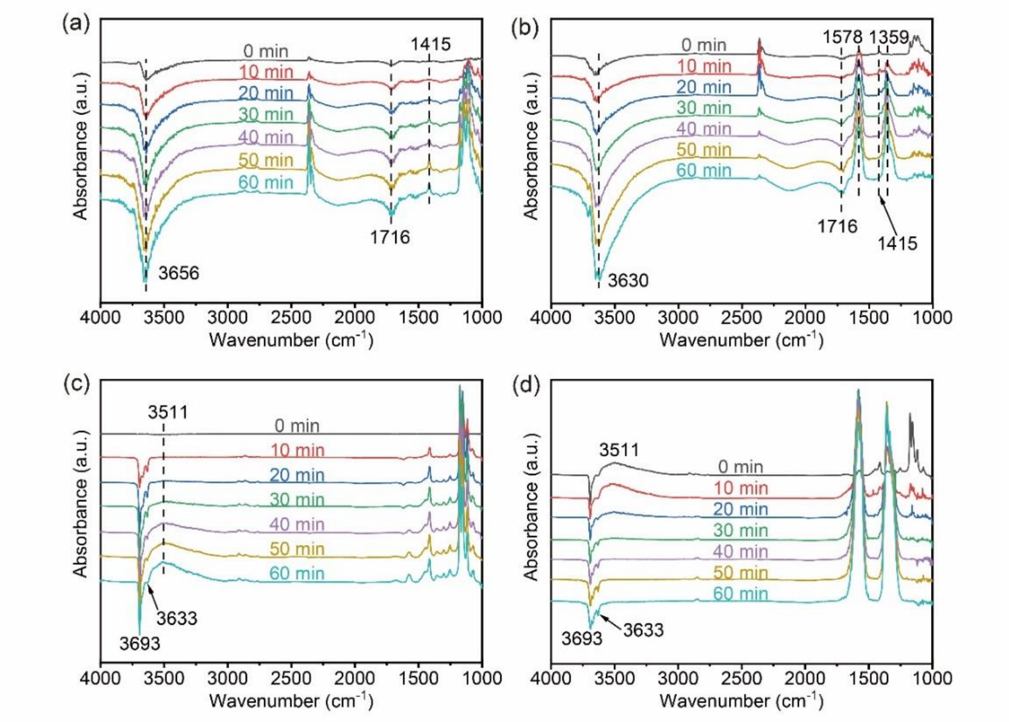


**Figure S12**. In situ DRIFT spectra of $TiO_2$ (a) before and (b) after illumination; $C_{60}$-EDA/$TiO_2$ (c) before and (d) after illumination.

**Table S1.** Comparisons of the catalytic performances between $C_{60}$-EDA/$TiO_2$ and other reported formaldehyde catalysts.

| Catalyst | Category | $m_{catalyst}$ | $V_{reactor}$ | $C_{i,HCHO}$ | $m_{HCHO}$ | $m_{HCHO}/m_{catalyst}$ | Removal Performance | Ref. |
|---|---|---|---|---|---|---|---|---|
| $C_{60}$-EDA/$TiO_2$ | sunlight | 0.5 g | 150 L | 136 mg/m$^3$ | 20.4 mg | 40.85 mg/g | $T_{50\%}$=50 min, $T_{90\%}$=195 min | This work |
| $TiO_2$/VACF | visible light | - | 72 L | 1 mg/m$^3$ | 0.072 mg | - | $T_{50\%}$=30 min, $T_{80\%}$=140 min | [1] |
| HMCN | sunlight | 0.2 g | 3 L | 6 mg/m$^3$ | 0.018 mg | 0.09 mg/g | $T_{50\%}$=30 min, $T_{75\%}$=105 min | [2] |
| $CeO_2$@LDHs | visible light | 10 g | 6.7 L | 32 mg/m$^3$ | 0.2144 | $1.44 \times 10^{-4}$ | $T_{50\%}$=105 min, $T_{87\%}$=300 | [3] |

| | | | | | | | | |
|---|---|---|---|---|---|---|---|---|
| | | | | | mg | mg/g | min | |
| GO/MnOx/CN | full light | 0.02 g | 0.5 L | 213.3 $mg/m^3$ | 0.1067 mg | 5.3 mg/g | $T_{50\%}$=2 min, $T_{90\%}$=12 min | [4] |
| g-$C_3N_4$-$TiO_2$/WZ | visible light | 0.15 g | 10 L | 4 $mg/m^3$ | 0.04 mg | 0.267 mg/g | $T_{50\%}$=50 min, $T_{90\%}$=300 min | [5] |
| $AgFeO_2$/g-$C_3N_4$ | visible light | 0.1 g | 0.8 L | 1.7 $mg/m^3$ | 0.0013 mg | 0.013 mg/g | $T_{50\%}$=250 min, $T_{87\%}$=540 min | [6] |
| C/CN-8 | visible light | 1 g | 20 L | 1200 $mg/m^3$ | 24 mg | 24 mg/g | $T_{85\%}$=300 min | [7] |
| $KC_3N_4$-3 | $\lambda$>420 nm | 0.1 g | 6 L | 400 $mg/m^3$ | 2.4 mg | 24 mg/g | $T_{50\%}$=7 min, $T_{90\%}$=24 min | [8] |
| 70CNCWO | full light | 0.05 g | - | - | 0.817 mg | 16.34 mg/g | $T_{50\%}$=60 min, $T_{90\%}$=160 min | [9] |
| $MoS_2$/g-$C_3N_4$ | visible light | 0.1 g | 0.5 L | 386.7 $mg/m^3$ | 0.193 mg | 1.93 mg/g | $T_{50\%}$=300 min, $T_{90\%}$=520 min | [10] |
| Cu/$C_3N_4$-3 | Visible light | 0.1 g | 6 L | 266.7 | 1.6 mg | 16 mg/g | $T_{50\%}$=25 min, $T_{90\%}$>70 min | [11] |
| GCN/$TiO_2$-x | Visible light | 0.25 g | - | 1.6 $mg/m^3$ | - | - | $T_{50\%}$>300 min | [12] |
| $Bi_2O_3$/$TiO_2$ | Visible light | 0.4 g | 216 L | 1.05 $mg/m^3$ | 0.2268 mg | 0.1225 mg/g | $T_{50\%}$=480 min, $T_{90\%}$=960 min | [13] |
| rGO/$TiO_2$ | Visible light | 0.2 g | 45 L | 0.67 $mg/m^3$ | 0.03 mg | 0.15 mg/g | $T_{50\%}$=76 min, $T_{90\%}$>240 min | [14] |
| Fe-nPAN | Visible light | 1 g | 0.5 L | 0.1 $mg/m^3$ | 0.00005 mg | $2.5 \times 10^{-8}$ mg/g | $T_{50\%}$=12 min, $T_{90\%}$=50 min | [15] |
| $MnO_2$/AlOOH | metal catalysts | 0.1 g | 5 L | 0.5 $mg/m^3$ | 0.0025 mg | $1.25 \times 10^{-4}$ mg/g | $T_{50\%}$=20 min, $T_{60\%}$=50 min | [16] |
| Pt/NiFe-LDH/rGO | noble metal catalysts | 0.1 g | 8.1 L | 266.7 $mg/m^3$ | 2.16 mg | 21.6 mg/g | $T_{50\%}$=8 min, $T_{90\%}$=55 min | [17] |

where $m_{catalyst}$ is the catalyst dosage, $V_{reactor}$ is the volume of reactor, $C_{i,HCHO}$ is the initial concentration of HCHO, $m_{HCHO}$ is the mass of HCHO, and $T_x$ is the time when the formaldehyde conversion rate is x, $m_{HCHO}/m_{catalyst}$ is the mass ratio of HCHO and catalyst, which represents the quality of degradable formaldehyde per unit catalyst. The higher the mass ratio, the better the catalytic performance of the catalyst.

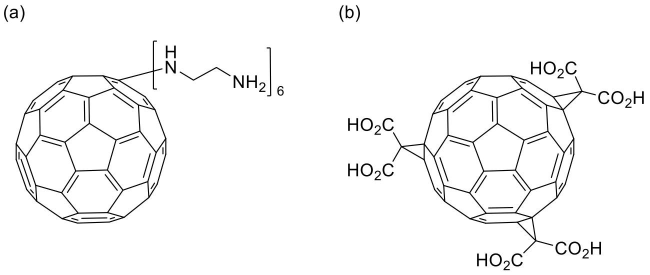


**Figure S13.** The molecular structures of the fullerene derivatives (a) $C_{60}$-EDA and (b) $C_{60}$-COOH.

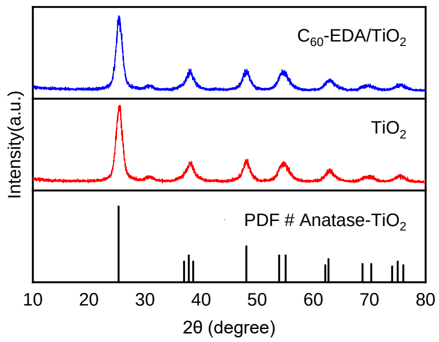


**Figure S14.** The XRD of $TiO_2$ and $C_{60}$-EDA/$TiO_2$ with comparison with standard PDF card of anatase titanium dioxide.

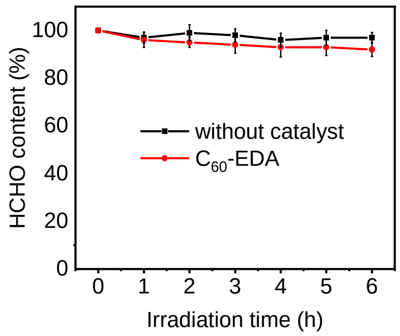


**Figure S15.** Catalytic performance without photocatalyst and with $C_{60}$-EDA.

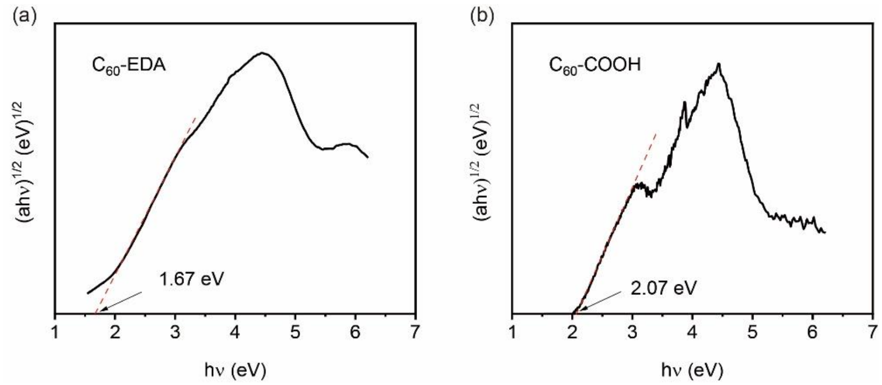


**Figure S16.** Band gap energy diagram of (a) $C_{60}$-EDA and (b) $C_{60}$-COOH.

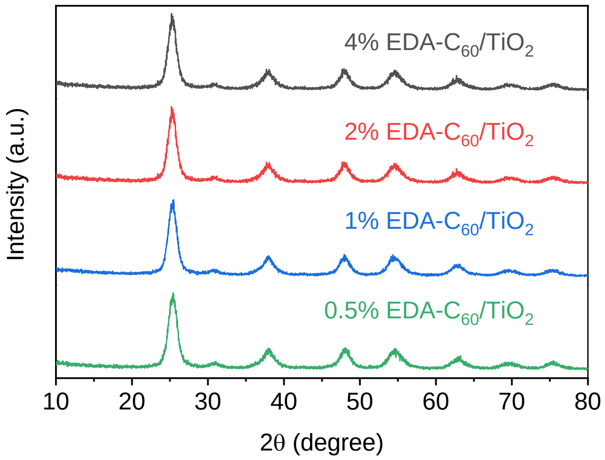


**Figure S17.** XRD spectra of the $C_{60}$-EDA/$TiO_2$ with different ratios of $C_{60}$-EDA.

# References


1. Yan, S.; Yu, J.; Zhu, B.; Qiao, K.; Cai, X.; Yuan, X.; Li, C. Photocatalytic Application of $TiO_2$ -Loaded Viscose-Based Activated Carbon Fibers Composite Catalyst: Degradation of Low Concentration Formaldehyde and Simultaneous Anti-Microbe. *ECS J. Solid State Sc.* **2021**, *10*, 011002–011012.
2. Chechia, H.; Wei-Fan, T.; Wei-Han, W.; Kun-Yi Andrew, L.; Miao-Ting, L.; Keizo, N. Hydroxylation and sodium intercalation on g-$C_3N_4$ for photocatalytic removal of gaseous formaldehyde. *Carbon* **2021**, *175*, 467–477.
3. Xia, S.J.; Zhang, G.H.; Meng, Y.; Yang, C.; Ni, Z.M.; Hu, J. Kinetic and mechanistic analysis for the photodegradation of gaseous formaldehyde by core-shell $CeO_2$@LDHs. *Appl. Catal. B-Environ.* **2020**, *278*, 119266–119278.
4. Wang, Z.; Yu, H.; Xiao, Y.; Zhang, L.; Guo, L.; Zhang, L.; Dong, X. Free-standing composite films of multiple 2D nanosheets: Synergetic photothermocatalysis/photocatalysis for efficient removal of formaldehyde under ambient condition. *Chem. Eng. J.* **2020**, *394*, 125014–125024.
5. Liu, S.-H.; Lin, W.-X. A simple method to prepare g-$C_3N_4$-$TiO_2$/waste zeolites as visible-light-responsive photocatalytic coatings for degradation of indoor formaldehyde. *J. Hazard. Mater.* **2019**, *368*, 468–476.
6. Tang, D.; Zhang, G. Fabrication of $AgFeO_2$/g-$C_3N_4$ nanocatalyst with enhanced and stable photocatalytic performance. *Appl. Surf. Sci.* **2017**, *391*, 415–422.
7. Li, X.; Qian, X.; An, X.; Huang, J. Preparation of a novel composite comprising biochar skeleton and "chrysanthemum" g-$C_3N_4$ for enhanced visible light photocatalytic degradation of formaldehyde. *Appl. Surf. Sci.* **2019**, *487*, 1262–1270.
8. Song, S.; Lu, C.; Wu, X.; Jiang, S.; Sun, C.; Le, Z. Strong base g-$C_3N_4$ with perfect structure for photocatalytically eliminating formaldehyde under visible-light irradiation. *Appl. Catal. B-Environ.* **2018**, *227*, 145–152.
9. Li, Y.; Wu, X.; Li, J.; Wang, K.; Zhang, G. Z-scheme g-$C_3N_4$@$Cs_xWO_3$ heterostructure as smart window coating for UV isolating, Vis penetrating, NIR shielding and full spectrum photocatalytic decomposing VOCs. *Appl. Catal. B-Environ.* **2018**, *229*, 218–226.
10. Lan, Z.; Yu, Y.; Yao, J.; Cao, Y. The band structure and photocatalytic mechanism of $MoS_2$-modified $C_3N_4$ photocatalysts

with improved visible photocatalytic Strong base g-$C_3N_4$ with perfect structure for photocatalytically activity. *Mater. Res. Bull.* **2018**, *102*, 433–439.

11. Liu, J.; Xiong, C.; Jiang, S.; Wu, X.; Song, S. Efficient evolution of reactive oxygen species over the coordinated π-delocalization g-$C_3N_4$ with favorable charge transfer for sustainable pollutant elimination. *Appl. Catal. B-Environ.* **2019**, *249*, 282–291.
12. Liu, S.-H.; Lin, W.-X. Heterostructured graphitic carbon nitride/titanium dioxide for enhanced photodegradation of low-concentration formaldehyde under visible light. *J. Photochem. Photobiol., A* **2019**, *378*, 66–73.
13. Huang, Q.; Wang, Q.; Tao, T.; Zhao, Y.; Wang, P.; Ding, Z.; Chen, M. Controlled synthesis of $Bi_2O_3/TiO_2$ catalysts with mixed alcohols for the photocatalytic oxidation of HCHO. *Environ. Technol.* **2019**, *40*, 1937–1947.
14. Yu, L.; Wang, L.; Sun, X.; Ye, D. Enhanced photocatalytic activity of rGO/$TiO_2$ for the decomposition of formaldehyde under visible light irradiation. *J. Environ. Sci.* **2018**, *73*, 138–146.
15. Han, X.; Han, Z.; Zhao, J.; Zhao, X. Photocatalytic degradation of formaldehyde by PAN nonwoven supported Fe(III) catalysts under visible light irradiation. *New J. Chem.* **2017**, *41*, 9380–9387.
16. Liu, Z.; Niu, J.; Long, W.; Cui, B.; Song, K.; Dong, F.; Xu, D. Highly Efficient $MnO_2$/AlOOH Composite Catalyst for Indoor Low-Concentration Formaldehyde Removal at Room Temperature. *Inorg. Chem.* **2020**, *59*, 7335–7343.
17. Wang, Y.; Jiang, C.; Le, Y.; Cheng, B.; Yu, J. Hierarchical honeycomb-like Pt/NiFe-LDH/rGO nanocomposite with excellent formaldehyde decomposition activity. *Chem. Eng. J.* **2019**, *365*, 378–388.
